# Age‐Related Parkinsonian Signs in Microdeletion 22q11.2

**DOI:** 10.1002/mds.28080

**Published:** 2020-05-09

**Authors:** Erik Boot, Thierry Q. Mentzel, Lisa D. Palmer, Peter N. van Harten, Connie Marras, Anthony E. Lang, Anne S. Bassett

**Affiliations:** ^1^ The Dalglish Family 22q Clinic University Health Network Toronto Ontario Canada; ^2^ Advisium,'s Heeren Loo Zorggroep Amersfoort The Netherlands; ^3^ Department of Psychiatry & Neuropsychology Maastricht University Maastricht The Netherlands; ^4^ Innova, Psychiatric Centre GGz Centraal Amersfoort The Netherlands; ^5^ The Edmond J. Safra Program in Parkinson's Disease and the Morton and Gloria Shulman Movement Disorders Clinic Toronto Western Hospital and University of Toronto Toronto Ontario Canada; ^6^ Division of Neurology, Department of Medicine University of Toronto Toronto Ontario Canada; ^7^ Clinical Genetics Research Program, and Campbell Family Mental Health Research Institute Centre for Addiction and Mental Health Toronto Ontario Canada; ^8^ Department of Psychiatry, Division of Cardiology, Department of Medicine, and Toronto General Research Institute University Health Network Toronto Ontario Canada; ^9^ Department of Psychiatry University of Toronto Toronto Ontario Canada

**Keywords:** 22q11.2 deletion syndrome, aging, Parkinson's disease, parkinsonism, wearable sensors

## Abstract

**Background:**

The recurrent hemizygous 22q11.2 deletion associated with 22q11.2 deletion syndrome has been identified as a genetic risk factor for early‐onset PD. However, little is known about early motor signs in this condition.

**Objectives:**

We examined the presence, severity and possible factors associated with parkinsonism in adults with 22q11.2 deletion syndrome and without PD.

**Methods:**

We compared motor signs between 82 adults with 22q11.2 deletion syndrome and 25 healthy controls, using the MDS‐UPDRS part III, and three‐dimensional motion‐tracker technology to quantify components of bradykinesia.

**Results:**

Median MDS‐UPDRS part III total and bradykinesia subscores were significantly higher in 22q11.2 deletion syndrome (median age: 26 years; range, 17–65) than in controls (*P* = 0.000; *P* = 0.000, respectively). Age was a significant contributor to bradykinesia subscore (*B* = 0.06; *P* = 0.01) and to the electronic bradykinesia component, velocity (*B* = –0.02; *P* = 0.000); psychotic illness did not significantly impact these analyses. In 22q11.2 deletion syndrome, MDS‐UPDRS–defined bradykinesia was present in 18.3%, rigidity in 14.6%, and rest tremor in 12.2%.

**Conclusions:**

Parkinsonian motor signs appear to be common and age related in 22q11.2 deletion syndrome. Longitudinal studies are needed to investigate possible symptom progression to PD. © 2020 The Authors. *Movement Disorders* published by Wiley Periodicals, Inc. on behalf of International Parkinson and Movement Disorder Society.

The 22q11.2 deletion associated with 22q11.2 deletion syndrome (22q11.2DS) has recently been identified as a genetic risk factor for early‐onset Parkinson's disease (PD).^1^, ^2^ The clinical and neuropathological characteristics of 22q11.2DS‐associated PD are comparable to those of idiopathic PD and some genetic forms of PD.^1^, ^3^ Little is known, however, about the prodromal stage of the disease and early motor signs in this high‐risk population.^4^ Also, there is some evidence that nondegenerative parkinsonian signs, including medication‐induced parkinsonism, may be common in 22q11.2DS at a relatively young age.^5^, ^6^, ^7^

In this study, we examined the presence and severity of parkinsonian motor signs in adults with 22q11.2DS, and demographic and clinical factors possibly associated with these signs, in comparison to healthy controls. We used the International Parkinson and Movement Disorder Society–sponsored revision of the UPDRS (MDS‐UPDRS),^8^ and three‐dimensional (3D) motion‐tracking technology to address the likelihood of low bradykinesia scores with limited variability that may be expected in a young population.^9^ We hypothesized that parkinsonian signs would be: (1) more common and more prominent in 22q11.2DS than in controls, (2) more severe in those with history of psychotic illness (mostly schizophrenia), and (3) positively correlated with increasing age in individuals with 22q11.2DS.

## Materials and Methods

This was a cross‐sectional study conducted at the Dalglish Family 22q Clinic, a 22q11.2DS specialty clinic at the Toronto General Hospital (Toronto, Ontario, Canada). Patients were recruited from a large 22q11.2DS cohort,^10^ and controls through patient's families and friends, and flyers posted at the hospital. We aimed at having a general match in distribution of age and sex between participants in both groups. This study was approved by the Research Ethics Boards of the University Health Network and Centre for Addiction and Mental Health, Toronto. Written informed consent was obtained from the participants and/or their substitute decision makers.

### Participants

Ninety‐two adults from 87 families with a chromosome 22q11.2 deletion, molecularly confirmed using standard methods,^10^ and 28 healthy controls entered the study. See the Supporting Information Supplementary Methods and Supporting Information Table S1 for details.

### Cognitive Functioning

The Montreal Cognitive Assessment (MoCA) was administered to all participants,^11^ as a proxy measure for cognitive functioning, given that full‐scale IQ (FSIQ) data were available only for a subsample. MoCA results showed a medium‐strong positive correlation with FSIQ (n = 48; r = 0.66; *P* = 0.000).

### Clinical Assessment of Parkinsonian Signs

Parkinsonian motor signs were assessed using the MDS‐UPDRS section III,^8^ by a physician (E.B.) experienced in movement disorders, and standard criteria were used for the presence/absence and laterality of parkinsonism (Supporting Information Supplementary Methods).^12^, ^13^

### Quantification of Bradykinesia Components

We quantified three different bradykinesia components (average [mean] cycle/stride velocity, amplitude, and duration), assessed using a 3D motion–tracker, as described previously (Supporting Information Supplementary Methods).^14^ All participants performed four repetitive motor tasks: (1) elbow flexion/extension; (2) lower arm pronation/supination; (3) leg agility (heel tap); and (4) gait. We computed composite z‐scores per separate bradykinesia component, using data from the combined tasks,^14^ in order to reduce the number of statistical tests.

### Statistical Analyses

Three sets of comparisons were performed including only those participants with complete data available, in order to be able to compare the results obtained from different tests: (1) adults with 22q11.2DS compared with controls; (2) a subsample of the 22q11.2DS group with no history of psychotic illness compared to controls, to eliminate the effect of psychotic illness on motor function^15^; and (3) within 22q11.2DS, comparing those with and without a history of psychotic illness. We used nonparametric tests to compare demographic data and MDS‐UPDRS scores, given the asymmetric data distribution. We used independent‐sample *t* tests to compare z‐scores. In 22q11.2DS, we used general linear models to examine the independent effect of possible demographic and clinical factors associated with severity of parkinsonism; age at assessment, cognitive function,^16^ history of psychotic illness,^15^ and sex. Pearson's correlation coefficients were used to consider the correlation between MDS‐UPDRS bradykinesia subscores and averaged z‐scores for velocity. General linear models were used to explore interactions between age and group on components of bradykinesia. All analyses were two‐tailed, with statistical significance defined as *P* < 0.05, using IBM SPSS software (Statistics 25; SPSS, Inc., Chicago, IL).

## Results

Demographic and clinical factors of the 107 participants (82 with 22q11.2DS and 25 healthy controls) who were included in the main analyses are presented in Table 1A. This cohort did not include 13 individuals, two excluded because of PD (both with 22q11.2DS)^3^, ^6^ and 11 because of incomplete or missing assessments (n = 8 for 22q11.2DS; n = 3 controls). By design, there were no significant differences in sex or age between the 22q11.2DS and control groups.

**Table 1A.** Demographic and clinical features and parkinsonian signs in adults with 22q11.2 deletion syndrome vs. healthy controls

	22q11.2 deletion syndrome (22q)						Healthy Controls (HC)		Analyses *P* a					
	Total 22q		22q‐NP Group		22q‐Psychosis Group^b^		Total HC						22q‐Psychosis vs. 22q‐NP	
	n = 82		n = 55		n = 27		n = 25		22q vs. HC		22q‐NP vs. HC			
**Demographics**	n	%	n	%	n	%	n	%		*P*		*P*		*P*
Male sex White Intellectual disability	43 65 46	52.4 79.3 56.1	28 45 28	50.9 81.8 50.9	15 20 18	55.6 74.1 66.7	10 23 n.a.	40.0 92.0 n.a.		0.36 0.23 —		0.47 0.32 —		0.82 0.56 0.24
	Median	Range (IQR)	Median	Range (IQR)	Median	Range (IQR)	Median	Range (IQR)		*P*		*P*		*P*
Age in years MoCA score	26 20	17 to 65 (20) 7 to 28 (6)	23 21	17 to 65 (18) 7 to 28 (6)	29 18	18 to 63 (19) 9 to 27 (9)	26 28	18 to 59 (14) 24 to 30 (3)		0.85 **0.000**		0.44 **0.000**		0.15 **0.005**
**Clinical assessments** c	Median	Range (IQR)	Median	Range (IQR)	Median	Range (IQR)	Median	Range (IQR)	Effect Size	*P*	Effect Size	*P*	Effect Size	*P*
MDS‐UPDRS part III scores Total (0–132) Bradykinesia (0–44) Rigidity (0–20) Rest tremor (0–20)	4 1 0 0	0 to 45 (7) 0 to 17 (2) 0 to 17 (0) 0 to 4 (0)	4 1 0 0	0 to 45 (6) 0 to 10 (1) 0 to 17 (0) 0 to 2 (0)	8 1 0 0	2 to 26 (9) 0 to 17 (2) 0 to 8 (0) 0 to 4 (0)	0 0 0 0	0 to 4 (1) 0 (0) 0 (0) 0 (0)	0.59 0.48 0.20 0.17	**0.000** **0.000** **0.04** 0.07	0.60 0.48 0.19 0.17	**0.000** **0.000** 0.09 0.12	0.35 0.29 0.15 0.15	**0.001** **0.008** 0.17 0.18
**Electronic assessments**	Mean	SD	Mean	SD	Mean	SD	Mean	SD	Cohen's *d*	*P*	Cohen's *d*	*P*	Cohen's *d*	*P*
Averaged z‐score Velocity Duration Amplitude	–0.25 0.21 –0.08	0.58 0.78 0.58	–0.27 0.20 –0.09	0.58 0.76 0.57	–0.22 0.21 –0.05	0.59 0.82 0.62	0.81 –0.63 0.26	0.52 0.36 0.48	1.47 1.08 0.59	**0.000** **0.000** **0.010**	1.44 1.08 0.61	**0.000** **0.000** **0.009**	0.09 0.01 0.16	0.75 0.97 0.74

Bold font indicates statistical significance.

a Fisher's exact tests for comparisons of categorical data, Mann‐Whitney U tests for clinical data, and independent‐samples t tests for data of electronic assessments. Effect sizes were determined by the z‐score, divided by the root of the total number of samples. Cohen's *d* effect sizes were determined by the mean difference between the groups, divided by the pooled SD. The significance did not change in any of the analyses when 3 adults on antipsychotic medication in the NP group were excluded.

b Twenty‐one persons in this group reported current antipsychotic use (including n = 2 with clozapine monotherapy).

c When we repeated the analyses including 7 more adults (n = 4 for 22q11.2DS; n = 3 controls) who only had MDS‐UPDRS data available, we found similar results (Supporting Information Table S2).

IQR, interquartile range; MoCA, Montreal Cognitive Assessment; n.a., not assessed; NP, no history of psychotic illness; Psychosis, history of a psychotic disorder; SD, standard deviation.

### Presence of Parkinsonian Motor Signs and Parkinsonism

Bradykinesia affected 15 (18.3%) adults with 22q11.2DS (median age: 39.0 years; range, 19–65). A significantly greater proportion with a psychotic illness had bradykinesia compared to those without such a history (33.3% vs. 10.9%; *P* = 0.03). Twelve (14.6%) persons with 22q11DS exhibited rigidity and 10 (12.2%) rest tremor, with proportions nonsignificantly greater in those with psychotic illness (22.2% vs. 10.9%; *P* = 0.20 and 18.5% vs. 9.1%; *P* = 0.29, respectively). Seven (8.5%; 5 with a psychotic illness) met criteria for parkinsonism (median age: 44.0 years; range, 19–65), including 2 with asymmetric parkinsonism.^13^, ^17^ In the control group, no bradykinesia, rigidity, or rest tremor was observed. Results were similar after including 7 more subjects (n = 4 for 22q11.2DS; n = 3 controls) with only MDS‐UPDRS data available (data not shown).

### Parkinsonian Motor Sign Assessments of Severity

Table 1A shows results for clinical (MDS‐UPDRS scores) and electronic assessments (composite z‐scores for velocity, amplitude, and duration). The 22q11.2DS group had significantly higher MDS‐UPDRS part III total scores, associated bradykinesia, and rigidity scores than controls; many adults had only slight‐to‐mild parkinsonian signs. Results for rigidity were nonsignificant if those with a psychotic illness were excluded. Effect sizes were weak (rigidity) to medium (total scores and bradykinesia subscores). Adults with 22q11.2DS and psychotic illness had significantly higher total and bradykinesia scores than those with no such history.

Composite z‐scores from the electronic assessments were significantly different between adults with 22q11.2DS and controls for all three components assessed, irrespective of psychotic illness, with mean z‐score differences 0.35 to 1.08 and medium (0.59) to very large (1.47) effect sizes. In 22q11.2DS, velocity was lower, duration longer, and amplitude smaller than in controls. Details for the separate motor tasks are provided in Supporting Information Tables S3 and S4.

### Relationship Between Age, Parkinsonian Signs, and Electronically Assessed Bradykinesia

**Figure 1:**
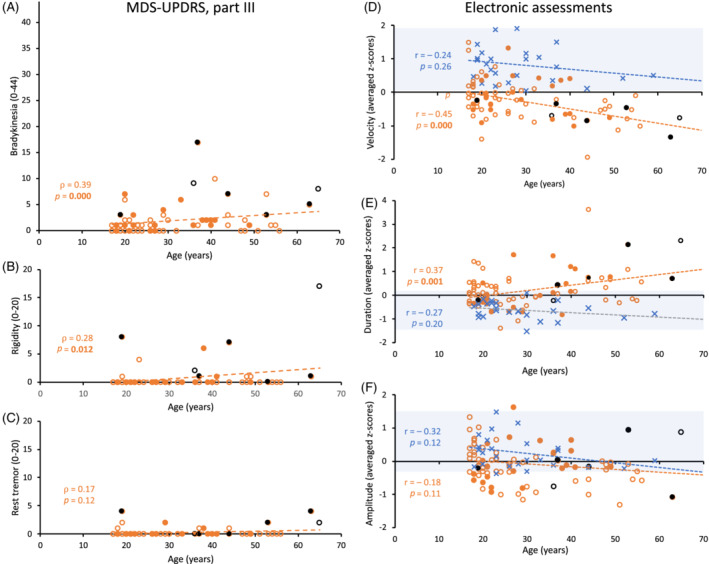
Scatterplots of the relationship between age and MDS‐UPDRS part III, scores (**A‐C**), and averaged standardized scores of cycle/stride velocity, duration, and amplitude as assessed with electronic 3D motion‐tracker technology (**D‐F**). Orange dot symbols indicate adults with 22q11.2DS. Black dot symbols indicate the 7 individuals meeting criteria for the presence of parkinsonism. Open dots (orange or black) indicate those with no history of psychotic illness, and filled dots those with such history. Blue ‘x’ symbols indicate healthy controls. (**A‐C**) None of the controls met MDS‐UPDRS criteria for bradykinesia, rigidity, or rest tremor; data not shown. Spearman's rank order correlations are shown to the left of the plots. (**D‐F**) Horizontal black lines represent the mean scores (z‐score=0) for the total study sample (see Supplementary Methods for details). Pale blue background indicates the range of results for the controls. We note, however, that in the absence of population‐based normative data, z‐scores were based on the total study sample, with the majority of participants having a 22q11.2 deletion. Pearson correlation coefficients are shown to the left of the plots. Linear regression models did not show a statistically significant interaction between age and study group (controls vs. 22q11.2DS) on the averaged standardized z‐scores, except for the bradykinesia component duration (*P *= 0.03). [Color figure can be viewed at wileyonlinelibrary.com]

Figure 1 shows the relationship at the individual level between age and (Fig. 1A–C) MDS‐UPDRS parkinsonian signs, significant for bradykinesia and rigidity in 22q11.2DS, and (Fig. 1D–F) motion‐tracker bradykinesia components. In 22q11.2DS, there was a statistically significant negative correlation of age with velocity (Fig. 1D), positive correlation with duration (Fig. 1E), and nonsignificant negative correlation with amplitude (Fig. 1F). The only statistically significant interaction between age and study group was for the duration component, with age having a negative effect in 22q11.2DS, but not in controls (*P* = 0.03). This suggests that in contrast to controls, in 22q11.2DS older individuals compensated relatively less for lower velocity with decreased amplitude than younger individuals, thus duration was longer.

### Demographic and Clinical Factors Associated With Parkinsonian Motor Sign Severity

The regression models to assess the association between demographic and clinical factors and MDS‐UPDRS Part III total and bradykinesia subscores in 22q11.2DS were statistically significant (*P* = 0.000 and *P* = 0.005), explaining 30.8% and 17.6% of the variance, respectively (Table 1B). Age explained 13.8% and cognitive function 6.3% of the variance of total scores. Only age made a statistically significant contribution to bradykinesia subscores, explaining 7.6% of the variance (Supporting Information Fig. S1). Results for psychotic illness were nonsignificant (Table 1B).

**Table 1B.** Associations between demographic and clinical factors, and severity of parkinsonian signs in 82 adults with 22q11.2 deletion syndrome^a^

	Standard Assessments of Parkinsonian Signs (MDS‐UPDRS part III)										Electronic Assessment (Velocity Component)				
	Total Score					Bradykinesia Subscore				
	*B*	*SE B*	β	*t*	*P*	*B*	*SE B*	β	*t*	*P*	*B*	*SE B*	β	*t*	*P*
Age Cognitive function Psychotic illness^b^^,^ c Male sex	0.22 –0.41 2.59 1.00	0.06 0.13 1.56 1.42	0.39 –0.27 0.17 0.07	3.93 –2.66 1.66 0.70	**0.000** **0.009** 0.10 0.48	0.06 –0.10 0.86 –0.39	0.02 0.06 0.65 0.60	0.28 –0.17 0.14 –0.07	2.66 –1.15 1.31 –0.66	**0.01** 0.13 0.19 0.51	–0.02 0.04 0.20 0.20	<0.01 0.01 0.12 0.11	–0.39 0.30 0.16 0.17	–4.04 3.00 1.62 1.80	**0.000** **0.004** 0.11 0.08

Bold font indicates statistical significance.

a Multiple regression analyses to examine the independent associations between demographic and clinical factors, and MDS‐UPDRS total scores, bradykinesia subscores (items 3.4–3.8 + 3.14), and the bradykinesia velocity component.

b When we altered the MDS‐UPDRS total score for a statistical outlier to just one unit larger than the next highest score to prevent an overproportional effect on the regression model, the values for psychotic illness changed to *B* = 3.31, *SE B* = 1.36, β = 0.24, *t* = 2.44, and *P* = 0.02.

c When we repeated the analyses for MDS‐UPDRS total scores leaving out cognitive function, the values for psychotic illness changed to *B* = 3.83, *SE B* = 1.55, β = 0.25, *t* = 2.48, and *P* = 0.02, suggesting a relationship between psychosis and cognitive function in 22q11.2DS as has been reported previously.^28^ When we repeated the analyses for bradykinesia subscores and electronic assessments leaving out cognitive function, the contribution of psychotic illness to the model did not reach statistical significance (*P* = 0.70 and *P* = 0.72, respectively), however.

*B*, unstandardized coefficient; *SE*, standard error; ß, standardized coefficient.

Similar to results for MDS‐UPDRS Part III total scores, the regression model for the electronically assessed bradykinesia component velocity was significant (*P* = 0.000), explaining 31.8% of the variance. Age and cognitive function were statistically significant contributors, explaining 14.9% and 7.5% of the variance, respectively; neither psychotic illness nor sex reached statistical significance (Table 1B). Remarkably, regression analysis results suggest that psychotic illness would contribute to increased, not decreased, velocity. The composite z‐score for velocity and MDS‐UPDRS bradykinesia subscore were weakly correlated (n = 82; r = –0.24; *P* = 0.03).

## Discussion

The results of this study involving a relatively young adult sample of 22q11.2DS are consistent with this genetic condition as a model for possible prodromal PD, with motor signs increasing with age. Although several decades earlier in 22q11.2DS, there are parallels with the emergence of age‐dependent parkinsonism in the general population,^9^, ^18^ where reported MDS‐UPDRS part III median (interquartile range; IQR) total scores were 3 (1.0–5.5) for 74 persons aged 72.2 (69.0–75.5) years in a higher‐risk subset and 1 (0.0–3.0) for a lower‐risk subset (n = 111) aged 64.9 (62.8–66.6) years.^18^ The findings are also consistent with those for other patient groups at increased risk of PD^19^, ^20^ and previous smaller studies of 22q11.2DS and healthy controls.^5^, ^6^, ^7^ Furthermore, the results indicate that vigilance for parkinsonian signs should not be restricted to adults with 22q11.2DS who take antipsychotic medication.^5^, ^6^, ^7^, ^21^

As expected,^15^ presence and severity of bradykinesia were greater in adults with than without a psychotic illness, but effect sizes were small to medium. In the regression analyses, the contributions of psychotic illness to bradykinesia outcomes were not statistically significant, perhaps related to insufficient effect size and/or sample size.

The results of the current study suggest that electronic assessments may be helpful in identification of subtle parkinsonian signs and/or to gain a better understanding of the course of the symptoms.^22^ Consideration may be given to electronic assessments in clinical settings,^22^ taking into account that they are objective and usually easy to learn,^14^ but focus only on one aspect of parkinsonism (ie, bradykinesia), while clinical rating scales are “gestalt based” and require greater expertise.

Cognitive function contributed significantly to MDS‐UPDRS total scores and bradykinesia component velocity. This would suggest involvement of similar circuits in these functions, as has also been postulated in idiopathic PD.^23^ Although the underlying causes of parkinsonism in 22q11.2DS remain to be determined, a multifactorial etiology is likely. In addition to emerging PD, alternative causes should be considered. These include, for example, basal ganglia calcifications,^24^ that may be secondary to hypoparathyroidism, a common manifestation of 22q11.2DS.^25^

The current study has several strengths, including the largest 22q11.2DS group with systematic assessments of parkinsonian signs to date, the first study to use electronic assessments, thereby allowing for detection of subtle bradykinesia, use of continuous measures, and comparisons of bradykinesia components,^26^ as well as elimination of inter‐rater effects by using standard assessments and a single rater. Potential methodological limitations include electronic assessments performed only with the dominant extremity, thereby missing contralateral bradykinesia, rater not blind to group status risking overcalling of signs in the psychosis subgroup, and absence of a qualitative motor assessment by a neurologist specialized in movement disorders. Design limitations include the restricted age range (none >65 years) also influencing the z‐scores in the absence of population‐based normative data, the relatively small control group, unavailability of neuroimaging data, and observational (cross‐sectional) nature of the study. Furthermore, not all adults with “parkinsonian signs” necessarily have true parkinsonism or PD.^20^, ^27^ Although causal inferences cannot be made, the results provide important input for future studies estimating causality.

The findings of this study indicate that age is a primary contributor to expression of bradykinesia in 22q11.2DS at a relatively young age in those without PD. Larger longitudinal studies, including neuroimaging,^28^ are needed to improve our knowledge of the course of symptoms, their relationship to underlying causes, and to psychotic disorders and treatments, in order to understand the risk profile of those with microdeletion 22q11.2 who go on to develop PD.

## Author Roles

(1) Research Project: A. Conception, B. Organization, C. Execution; (2) Statistical Analysis: A. Design, B. Execution, C. Review and Critique; (3) Manuscript Preparation: A. Writing of the First Draft, B. Review and Critique.

E.B.: 1A, 1B, 1C, 2A, 2B, 3A

T.M.: 1A, 1B, 3B

L.P.: 1B, 1C, 3B

P.H.: 1A, 3B

C.M.: 1A, 3B

A.L.: 1A, 3B

A.S.B.: 1A, 1B, 2A, 2C, 3B

## Financial Disclosures

C.M. and A.E.L. receive grant support from the Michael J. Fox Foundation, the Canadian Institutes of Health Research (CIHR), and the Parkinson's Foundation. C.M. also receives grant support from the International Parkinson and Movement Disorder Society and the National Institutes of Health (NIH). She has served as a consultant for Acorda Therapeutics and has received honoraria for teaching from EMD Serono and for participating as a Steering Committee member from the Michael J. Fox Foundation. She has served on the advisory board for Denali Therapeutics. A.E.L. has served as a consultant for AbbVie, AFFiRis, Biogen, Janssen, Lilly, Lundbeck, Merck, Paladin, Roche, Sun Pharma, Theravance, and Corticobasal Degeneration Solutions. He has served on advisory boards for Jazz Pharma, PhotoPharmics, and Sunovion and received honoraria from Sun Pharma, AbbVie, and Sunovion. A.E.L. has also received grant support from Brain Canada, Corticobasal Degeneration Solutions, the Edmond J. Safra Philanthropic Foundation, the Ontario Brain Institute, Parkinson Canada, and W. Garfield Weston Foundation and royalties from Elsevier, Saunders, Wiley‐Blackwell, Johns Hopkins Press, and Cambridge University Press. A.S.B. holds the Dalglish Chair in 22q11.2 Deletion Syndrome and has received grant support from CIHR (MOP‐53216, MOP‐313331, and PJT‐148924), NIMH (5U01MH101723), and the University of Toronto McLaughlin Centre.

## Supporting information


**Appendix** **S1:** Supplementary Material
