# Supplementary material for: Age‐Related Parkinsonian Signs in Microdeletion 22q11.2
Source: Mov Disord. 2020 May 9;35(7):1239–45. doi: 10.1002/mds.28080 (PMC7497092; doi:10.1002/mds.28080)
Supplement: Supplementary file 1 — Appendix S1: Supplementary Material [file MDS-35-1239-s001.doc]

**Supplementary Methods**

***Participants***

For the 22q11.2 group, recruitment for the originating cohort was through three main sources, as previously described, from most to least frequent: a congenital cardiac disease clinic, medical genetics, and psychiatry;[^1^](#_ENREF_1) none were from neurology. Patients had been followed clinically for up to 20+ years by specialists in 22q11.2DS.[^2-4^](#_ENREF_2) The deletion was typical (overlapping the LCR22A-LCR22B region) in all but one case (overlapping the LCR22B-LCR22D region).[^5^](#_ENREF_5) The 22q11.2 deletion origin was *de novo* in 69 (75.0%), inherited in 11 (13.0%), and uncertain in another 11 cases. We excluded patients with Parkinson’s disease (PD), defined as a clinical diagnosis by a neurologist, including bradykinesia and at least one of either rest tremor or rigidity,[^6^](#_ENREF_6) from the analyses, since for this study we were especially interested to gain insight into non-degenerative parkinsonian signs and prodromal signs of the disease, rather than disease progression. Additional exclusion criteria for healthy controls were: 1) a suspected 22q11.2 deletion as judged by a physician experienced in 22q11.2 deletion syndrome (22q11.2DS), 2) intellectual disability (ID) or otherwise unable to give informed consent, 3) current or past history of psychiatric disorder as assessed using the Mini International Neuropsychiatric Interview, sixth edition (MINI-6.0.0),[^7^](#_ENREF_7) 4) previously diagnosed neurological disorder, or 5) a history of any dopaminergic medication. The presence or absence, and the severity, of ID, as well as psychiatric diagnoses, were determined by a physician specialized in ID medicine (E.B.), and a psychiatrist (A.S.B.) according to the Diagnostic and Statistical Manual of Mental Disorders, fifth edition (DSM-5), based on results from the Structured Clinical Interview for Diagnostic and Statistical Manual of Mental Disorders, fourth Edition (SCID-IV), in addition to all information obtained from direct observation, collateral history from family members, and available information from lifetime medical records.[^8^](#_ENREF_8) Fifteen (16.3%) had normal, and 24 (26.1%) borderline, intellectual functioning. Forty-eight (52.2%) had a mild, and 5 (5.4%) a moderate ID. Thirty-four individuals (37.0%) had a lifetime diagnosis of a psychotic disorder (schizophrenia, n=18; schizoaffective disorder, n=4; other psychotic disorder, n=12; median age at onset 20 (range 12-57) years and 58 (63.0%) had no history of psychotic illness. We calculated chlorpromazine equivalents to compare antipsychotic medication doses, using linear regression conversion formulas if available.[^9^](#_ENREF_9) We applied alternative methods for methotrimeprazine and zuclopenthixol,[^10^](#_ENREF_10) and lurasidone and asenapine.[^11^](#_ENREF_11) We also calculated an adjusted (10% of the calculated)[^9^](#_ENREF_9) value in participants with 22q11.2DS who used clozapine to prevent an over proportional effect of antipsychotic dose, as clozapine is not expected to cause motor side effects. Three other participants with no history of psychotic illness reported current low dose antipsychotic medication use (indications: tic disorder, insomnia, and unclear, respectively). Eight participants with psychotic illness reported no current antipsychotic use. One adult with a 22q11.2 deletion (*de novo*) had a first-degree family member with history of PD (estimated age at onset 51 years), and another (also *de novo* 22q11.2 deletion) was known to have a parent with laryngeal dystonia (estimated age at onset 40 years). In the 22q11.2DS group, four affected participating adults had participating adult offspring, and there was one brother-brother pair. Two healthy controls, where a 22q11.2 deletion was ruled out by molecular testing, had a participating affected sibling with a *de novo* 22q11.2 deletion.

***Criteria for the presence or absence of (asymmetric) parkinsonism***

As part of the assessment of parkinsonian motor signs using the MDS-UPDRS section III by a physician experienced in movement disorders who had completed the MDS-UPDRS Training Program and Exercise (<https://www.movementdisorders.org/>), we used standard criteria for the presence/absence of bradykinesia: score of ≥1 in 2 motor tests on the same side of the body or a score of ≥2 in 1 motor test (items 3.4-3.8); rest tremor, score of ≥1 (item 3.17); rigidity, score ≥1 (item 3.3).[^12^](#_ENREF_12) For each side of the body, the sum of the scores was calculated; bradykinesia: 3.4-3.8, rigidity: 3.3, and tremor: 3.15-3.17. We defined asymmetric parkinsonism as either an asymmetric index (higher sum – lower sum)/(higher sum1lower sum) ≥0.20 or a side difference of MDS-UPDRS total motor score ≥4,[^13^](#_ENREF_13) for the n=7 with parkinsonism.

***Electronic assessment of bradykinesia components***

We assessed the degree of different components of bradykinesia using a wireless 3D human motion tracker with five inertial sensors (MTw; Xsens, Enschede, the Netherlands), as described previously.[^14^](#_ENREF_14) We attached the sensors to the subject’s dominant upper and lower arm and leg, and waist, with velcro straps. The sensors sent the crude data via the Awinda radio protocol (Xsens) to a laptop using Matlab (version 2015b, Mathworks) and the Xsens SDK (version 4.3). All participants performed four repetitive motor tasks: 1) elbow flexion/extension, 2) lower arm pronation/supination, 3) leg agility (heel tap), and 4) gait. We instructed participants to perform the first three tasks for 25 seconds, and motivated them to keep going after approximately 15 seconds. We also instructed them to perform the first two tasks as fast and as wide as possible, and to raise their foot about 5-10 centimeter above the ground with the heel tap. For the gait task, we instructed the participants to walk 20 meters in total at a normal to brisk pace, and to turn around and walk back after passing a marker on the floor (every 5 meters, 4 turns). We instructed all participants both with verbal instruction and by demonstrating each task. These electronic data were initially processed and analyzed as described previously.[^15^](#_ENREF_15) We subsequently computed composite z-scores per separate bradykinesia component, using data from the combined tasks,[^14^](#_ENREF_14) in order to reduce the number of statistical tests. Composite scores were calculated by converting the separate scores on the tasks to z-scores based on the mean and standard deviation of the total study sample given the relatively small sample size and absence of reference data, and averaging the summed z-scores.[^14^](#_ENREF_14) Composite z-scores were calculated for those participants with data for at least two of the four tasks and additionally, in 22q11.2DS, all data available to examine the independent effect of demographic and clinical factors that possibly associated with bradykinesia. For this specific analysis, we used the component velocity, as velocity outcomes explained most of the variance on clinical bradykinesia in previous research using this instrument.[^14^](#_ENREF_14)

***Statistical analyses***

Primary analyses were performed including only those participants with complete clinical and electronic assessment data available (i.e., both MDS-UPDRS scores (items 3.4-3.8 + 3.14) as well as a composite z-score). We repeated the analyses including all participants with data available for a specific test. In adults with 22q11.2DS, we used general linear models to examine the independent effect of possible demographic and clinical factors associated with severity of parkinsonism. The variables considered were age at assessment, cognitive function,[^16^](#_ENREF_16) history of psychotic illness,[^17^](#_ENREF_17) and sex. Assumptions of multiple regression, including linearity, were tested to ensure no violations: Cook’s D was <1 in all cases, suggesting the absence of influential observations, and variance inflation values were <2 in all cases, suggesting no issues with multicollinearity.

**References**

1. Bassett AS, Chow EW, Husted J, et al. Clinical features of 78 adults with 22q11 deletion syndrome. Am J Med Genet A 2005;138(4):307-313.

2. Butcher NJ, Boot E, Lang AE, et al. Neuropsychiatric expression and catatonia in 22q11.2 deletion syndrome: An overview and case series. Am J Med Genet A 2018;176(10):2146-2159.

3. Malecki SL, Van Mil S, Graffi J, et al. A genetic model for multimorbidity in young adults. Genet Med 2020;22(1):132-141.

4. Van L, Heung T, Graffi J, et al. All-cause mortality and survival in adults with 22q11.2 deletion syndrome. Genet Med 2019;21(10):2328-2335.

5. McDonald-McGinn DM, Sullivan KE, Marino B, et al. 22q11.2 deletion syndrome. Nat Rev Dis Primers 2015;1:15071.

6. Postuma RB, Berg D, Stern M, et al. MDS clinical diagnostic criteria for Parkinson's disease. Mov Disord 2015;30(12):1591-1601.

7. Sheehan DV, Lecrubier Y, Sheehan KH, et al. The Mini-International Neuropsychiatric Interview (M.I.N.I.): the development and validation of a structured diagnostic psychiatric interview for DSM-IV and ICD-10. J Clin Psychiatry 1998;59 Suppl 20:22-33;quiz 34-57.

8. Fiksinski AM, Breetvelt EJ, Lee YJ, et al. Neurocognition and adaptive functioning in a genetic high risk model of schizophrenia. Psychol Med 2019;49(6):1047-1054.

9. Andreasen NC, Pressler M, Nopoulos P, Miller D, Ho BC. Antipsychotic dose equivalents and dose-years: a standardized method for comparing exposure to different drugs. Biol Psychiatry 2010;67(3):255-262.

10. Gardner DM, Murphy AL, O'Donnell H, Centorrino F, Baldessarini RJ. International consensus study of antipsychotic dosing. Am J Psychiatry 2010;167(6):686-693.

11. Leucht S, Samara M, Heres S, Patel MX, Woods SW, Davis JM. Dose equivalents for second-generation antipsychotics: the minimum effective dose method. Schizophr Bull 2014;40(2):314-326.

12. Adler CH, Beach TG, Hentz JG, et al. Low clinical diagnostic accuracy of early vs advanced Parkinson disease: clinicopathologic study. Neurology 2014;83(5):406-412.

13. Ham JH, Lee JJ, Kim JS, Lee PH, Sohn YH. Is dominant-side onset associated with a better motor compensation in Parkinson's disease? Mov Disord 2015;30(14):1921-1925.

14. Mentzel TQ, Lieverse R, Levens A, et al. Reliability and validity of an instrument for the assessment of bradykinesia. Psychiatry Res 2016;238:189-195.

15. Mentzel TQ, Mentzel CL, Mentzel SV, Lieverse R, Daanen HA, van Harten PN. Instrumental Assessment of Bradykinesia: A Comparison Between Motor Tasks. IEEE J Biomed Health Inform 2016;20(2):521-526.

16. Fervaha G, Agid O, Takeuchi H, et al. Extrapyramidal symptoms and cognitive test performance in patients with schizophrenia. Schizophr Res 2015;161(2-3):351-356.

17. Walther S, Mittal VA. Motor system pathology in psychosis. Curr Psychiatry Rep 2017;19(12):97.

**Supplementary Table S1.** Demographic and clinical features of all participants that entered the study (n=120)

|  | 22q11.2 deletion syndrome | | | | | | Healthy controls | | Analyses *P* ^a^ | | |
| --- | --- | --- | --- | --- | --- | --- | --- | --- | --- | --- | --- |
|  | Total 22q  n=92 | | NP group  n=58 | | Psychosis group  n=34 | | Total HC  n=28 | | 22q *vs* HC | 22q-NP *vs* HC | 22q-psychosis *vs* 22q-NP |
|  | n | % | n | % | n | % | n | % | *P* | *P* | *P* |
| Male sex | 47 | 51.1 | 28 | 48.3 | 19 | 55.9 | 12 | 42.9 | 0.52 | 0.65 | 0.52 |
| Caucasian | 72 | 78.3 | 47 | 81.0 | 25 | 73.5 | 25 | 89.3 | 0.28 | 0.53 | 0.44 |
| Intellectual disability | 53 | 57.6 | 31 | 53.4 | 22 | 64.7 | n.a. | n.a. | - | - | 0.38 |
|  | Median | Range (IQR) | Median | Range (IQR) | Median | Range (IQR) | Median | Range (IQR) |  |  |  |
| Age in years | 26.0 | 17-65 (20) | 23.5 | 17-65 (23) | 33.0 | 18-63 (19) | 27.0 | 18-61 (14) | 0.87 | 0.39 | 0.12 |
| MoCA score ^b^ | 20.0 | 7-28 (7) | 21.0 | 7-28 (6) | 17.5 | 7-27 (10) | 28.0 | 23-30 (3) | **0.000** | **0.000** | **0.009** |

Abbreviations: Total 22q = Total 22q11.2 deletion syndrome sample, NP = no history of psychotic illness, Psychosis = history of a psychotic disorder, HC = healthy controls, n.a. = not assessed, IQR = interquartile range, MoCA = Montreal Cognitive Assessment.

^a^ *P*-values from Fisher’s exact tests for comparisons of sex, ethnicity, and the presence of intellectual disability, and Mann-Whitney U tests for age and MoCA score comparisons. Bold font indicates statistically significant *P*-values.

^b^ MoCA scores available as a proxy for cognitive functioning for n=90 (n=32 with psychotic illness) participants with 22q11.2DS and all 28 healthy controls.

**Supplementary Table S2. MDS-UPDRS scores of all participants with available data (n=114)**

|  | 22q11.2 deletion syndrome | | | | | | Healthy controls | | Analyses *P* ^a^ | | |
| --- | --- | --- | --- | --- | --- | --- | --- | --- | --- | --- | --- |
| Clinical assessments | Total 22q  n=86 ^b^ | | NP group  n=57 | | Psychosis group n=29 | | Total HC  n=28 | | 22q  *vs* HC | 22q-NP  *vs* HC | 22q-psychosis *vs* 22q-NP |
|  | Median | Range (IQR) | Median | Range (IQR) | Median | Range (IQR) | Median | Range (IQR) | *P* | *P* | *P* |
| MDS-UPDRS, total score (0-132)  Bradykinesia score (0–44)  Rigidity score (0–20)  Rest tremor score (0–20) | 5  1  0  0 | 0-45 (7)  0-17 (2)  0-17 (0)  0-4 (0) | 4  1  0  0 | 0-45 (6)  0-10 (1)  0-17 (0)  0-4 (0) | 8  1  0  0 | 2-26 (9)  0-17 (3)  0-8 (0)  0-4 (0) | 0  0  0  0 | 0-4 (1)  0 (0)  0 (0)  0 (0) | **0.000**  **0.000**  **0.03**  **0.048** | **0.000**  **0.000**  0.06  0.08 | **0.002**  **0.01**  0.30  0.34 |

Abbreviations: Total 22q = Total 22q11.2 deletion syndrome sample, NP = no history of psychotic illness, Psychosis = history of psychotic illness, HC = healthy controls, IQR = interquartile range, MDS-UPDRS = Movement Disorder Society-sponsored revision of the Unified Parkinson's Disease Rating Scale (part III).

^a^ *P*-values from Mann-Whitney U for group comparisons on the MDS-UPDRS. Bold font indicates statistically significant *P*-values. The significance did not change in any of the analyses when three adults on antipsychotic medication were excluded.

^b^ Of the 92 adults with 22q11.2DS, 6 (of whom 5 with history of psychotic illness) were excluded from the analyses or had no data due to Parkinson’s disease (n=2), active psychotic symptoms (n=3), or lack at time at the study day (n=1).

**Supplementary Table S3. Electronic assessments of bradykinesia components (velocity, duration, and amplitude) in 82 adults with 22q11.2 deletion syndrome and 25 healthy controls**

|  | 22q11.2 deletion syndrome | | | | | | Healthy controls | | Analyses ^b^ | | | | | |
| --- | --- | --- | --- | --- | --- | --- | --- | --- | --- | --- | --- | --- | --- | --- |
|  | Total 22q  Max n=82 | | 22q-NP  Max n=55 ^a^ | | 22q-Psychosis  Max n=27 | | Total HC  Max n=25 | | Total 22q  *vs* HC | | 22q-NP  *vs* HC | | 22q-psychosis *vs* 22q-NP | |
|  | Mean | SD | Mean | SD | Mean | SD | Mean | SD | Effect size | *P* | Effect size | *P* | Effect size | *P* |
| Upper body ^c^  Flex-/extension  Velocity, deg/s  Duration, s  Amplitude, deg  Pro-/supination  Velocity, deg/s  Duration, s  Amplitude, deg | 258.8  0.8  94.6  349.1  0.5  80.2 | 80.6  0.2  28.5  97.1  0.1  21.4 | 256.7  0.8  93.6  356.0  0.5  81.8 | 88.0  0.2  29.5  97.8  0.1  21.4 | 262.9  0.8  96.6  335.5  0.5  77.1 | 64.3  0.3  27.0  96.1  0.2  21.3 | 360.8  0.5  84.3  431.2  0.4  91.9 | 87.4  0.1  23.9  132.7  0.1  22.3 | 1.10  1.20  0.37  0.74  0.83  0.53 | **0.000**  **0.000**  0.10  **0.007**  0.06  **0.02** | 1.04  1.25  0.37  0.65  1.11  0.46 | **0.000**  **0.000**  0.17  **0.006**  0.14  0.06 | 0.07  0.02  0.11  0.21  0.13  0.22 | 0.75  0.93  0.65  0.38  0.65  0.36 |
| Lower body ^d^  Leg agility  Velocity, deg/s  Duration, s  Amplitude, deg  Gait  Velocity, m/s  Duration, s  Amplitude, m | 66.1  0.5  15.3  1.1  1.1  1.2 | 25.4  0.1  7.1  0.2  0.1  0.2 | 66.1  0.5  15.3  1.1  1.1  1.2 | 25.6  0.1  6.8  0.2  0.1  0.2 | 65.9  0.5  15.4  1.2  1.1  1.2 | 25.5  0.1  7.9  0.2  0.1  0.2 | 98.9  0.4  17.4  1.4  1.0  1.4 | 20.2  0.1  3.7  0.2  0.1  0.2 | 1.17  0.83  0.32  1.30  1.00  1.05 | **0.000**  **0.000**  0.06  **0.000**  **0.000**  **0.000** | 1.15  0.77  0.34  1.25  1.00  1.05 | **0.000**  **0.000**  0.08  **0.000**  **0.000**  **0.000** | 0.01  0.09  0.01  0.53  0.10  0.23 | 0.98  0.70  0.94  0.43  0.69  0.34 |

Abbreviations: Total 22q = Total 22q11.2 deletion syndrome sample, NP = no history of psychotic illness, Psychosis = history of a psychotic disorder, HC = healthy controls, SD = standard deviation, s = second, deg = degree, m = meter.

^a^ The significance did not change when three adults on antipsychotic medication were excluded from analyses, except that we found significant between-group differences in amplitude between total 22q *vs* HC with the leg agility task (*P*=0.039), and NP *vs* HC with the pronation/supination and leg agility task (both *P*=0.042).

^b^ *P*-values from Mann-Whitney U Tests. Bold font indicates statistically significant *P*-values. Cohen’s d effect sizes were determined by calculating the mean difference between the groups, divided by the pooled standard deviation.

^c^ Data calculated from sensors on upper and lower dominant arm.

^d^ Data calculated from sensors on upper and lower dominant leg with [gait task] or without [leg agility task] the sensor on waist.

**Supplemantary Table S4. Electronic assessments of bradykinesia components (velocity, duration, and amplitude) in all participants with data available**

|  | 22q11.2 deletion syndrome | | | | | | Healthy controls | | Analyses ^c^ | | | | | |
| --- | --- | --- | --- | --- | --- | --- | --- | --- | --- | --- | --- | --- | --- | --- |
|  | Total 22q  Max n=84 ^a^ | | 22q-NP  Max n=56 ^b^ | | 22q-Psychosis  Max n=28 | | Total HC  Max n=27 ^a^ | | Total 22q  *vs* HC | | 22q-NP  *vs* HC | | 22q-psychosis *vs* 22q-NP | |
|  | Mean | SD | Mean | SD | Mean | SD | Mean | SD | Effect size | *P* | Effect size | *P* | Effect size | *P* |
| Upper body ^d^  Flex-/extension ^#^  Velocity, deg/s  Duration, s  Amplitude, deg  Pro-/supination ^*^  Velocity, deg/s  Duration, s  Amplitude, deg | 260.8  0.8  95.3  347.6  0.5  80.0 | 81.0  0.2  28.6  97.4  0.1  21.3 | 257.4  0.8  94.3  356.0  0.5  81.8 | 87.3  0.2  29.7  97.8  0.1  21.4 | 267.5  0.8  97.4  331.7  0.5  76.6 | 67.6  0.3  26.8  96.4  0.2  21.1 | 366.5  0.5  85.8  431.2  0.4  91.9 | 90.4  0.1  24.7  132.7  0.1  22.3 | 1.12  1.20  0.34  0.59  0.83  0.54 | **0.000**  **0.000**  0.13  **0.006**  0.05  **0.02** | 1.07  1.25  0.30  0.65  1.11  0.46 | **0.000**  **0.000**  0.21  **0.006**  0.14  0.06 | 0.12  0.01  0.11  0.25  0.15  0.24 | 0.43  0.69  0.34  0.29  0.60  0.30 |
| Lower body ^e^  Leg agility ^§^  Velocity, deg/s  Duration, s  Amplitude, deg  Gait ^  Velocity, m/s  Duration, s  Amplitude, m | 66.1  0.5  15.4  1.1  1.1  1.2 | 25.1  0.1  7.1  0.2  0.1  0.2 | 66.2  0.5  15.3  1.1  1.1  1.2 | 25.4  0.1  6.8  0.2  0.1  0.2 | 65.9  0.5  15.5  1.2  1.1  1.2 | 25.0  0.1  7.7  0.2  0.1  0.2 | 98.9  0.4  17.4  1.4  1.0  1.3 | 20.2  0.1  3.7  0.3  0.1  0.3 | 1.18  0.83  0.31  1.30  1.00  0.53 | **0.000**  **0.000**  0.07  **0.000**  **0.005**  **0.004** | 1.16  0.77  0.37  1.30  1.00  0.52 | **0.000**  **0.000**  0.09  **0.000**  **0.000**  **0.000** | 0.01  0.08  0.03  0.56  0.10  0.24 | 0.96  0.73  0.94  0.43  0.69  0.34 |

Abbreviations: Total 22q = Total 22q11.2 deletion syndrome sample, NP = no history of psychotic illness, Psychosis = history of a psychotic disorder, HC = healthy controls, SD = standard deviation, s = second, deg = degree, m = meter.

^a^ Participants with no data for a specific task were excluded from that specific analysis due to ^#^ a technical issue (n=1 HC), ^*^ arthritis, and pain related to a previous fracture and surgery, respectively (n=3 NP) and a technical issue (n=2 HC), ^§^ a clubfoot (n=1 NP) and a technical issue (n=2 HC), ^ spasticity in the dominant hand (n=1 NP), a clubfoot (n=1 NP, impaired mobility (n=1 NP), and a technical issue (n=4 NP, n=1 HC).

^b^ The significance did not change in any of the between-group comparisons when three adults with no history of psychotic illness on antipsychotic medication were excluded from analyses, except that we found a significant between-group difference in amplitude between NP *vs* HC with the pronation/supination task (*P*=0.048).

^c^ *P*-values from Mann-Whitney U Tests. Bold font indicates statistically significant *P*-values. Cohen’s d effect sizes were determined by calculating the mean difference between the groups, divided by the pooled standard deviation.

^d^ Data calculated from sensors on upper and lower dominant arm.

^e^ Data calculated from sensors on upper and lower dominant leg with [gait task] or without [leg agility task] the sensor on waist.
